# Comparing the effects of analogical instruction with internal and external focus of attention on golf putting skill learning in children with developmental coordination disorder: Emphasizing mental representation structure

**DOI:** 10.1371/journal.pone.0351065

**Published:** 2026-06-16

**Authors:** Saeed Nazari Kakvandi, Hesam Ramezanzade, Hassan Kordi

**Affiliations:** 1 Department of Motor Behavior, Faculty of Physical Education and Sport Sciences, Ferdowsi University of Mashhad, Mashhad, Iran; 2 Department of Sport Sciences, School of Humanities, Damghan University, Damghan, Iran; 3 Department of Behavioral and Cognitive Sciences in Sport, Faculty of Sport Sciences and Health, University of Tehran, Tehran, Iran; Virginia Tech: Virginia Polytechnic Institute and State University, UNITED STATES OF AMERICA

## Abstract

This study examined the effects of implicit learning-based instruction—analogy, external focus (EF), and their combination—on golf putting performance and learning in children with developmental coordination disorder (DCD). Given that internal focus of attention often competes with external focus by drawing attention to body movements, combining analogy with EF may help direct attention externally by simplifying movement execution. Sixty children aged 7–9 with DCD were randomly assigned to five groups: analogical instruction, EF, internal focus (IF), analogical-external focus (A-EF), and control. The IF group focused on their striking hand; the EF group focused on the putter’s path and target. The analogical group performed a pendulum-like swing based on a biomechanical metaphor. The A-EF group combined the pendulum analogy with EF instructions. The control group received no instructional cues. Participants completed a pretest, followed by three acquisition sessions (3 × 20 trials per session), immediate and delayed retention tests (72 hours post-practice), a transfer test (from a 5.5-meter distance), and an automaticity test under dual-task conditions. Results showed that, collapsed across acquisition sessions, both the external focus (EF) and combined analogy-external focus (A-EF) groups significantly outperformed the internal focus (IF) group. In retention, transfer, and dual-task performance, all three implicit learning groups (A, EF, A-EF) performed significantly better than the IF and control groups. These findings suggest that implicit instructional methods, particularly the combined approach, enhance motor learning in children with DCD, likely due to reduced reliance on working memory and greater automaticity of movement execution.

## Introduction

Motor learning, defined as relatively stable changes in the capability to perform motor tasks resulting from practice or experience, is especially critical during childhood, when fundamental skills such as running, jumping, and throwing are acquired and refined. These foundational skills enable the development of more complex motor abilities essential for sports and daily physical activities [1]. However, this developmental trajectory is not universal. Some children face persistent challenges in acquiring motor skills, notably those diagnosed with Developmental Coordination Disorder (DCD), a neurodevelopmental condition marked by significant impairments in motor coordination that interfere with daily functioning and academic performance [2].

Children with DCD exhibit notable deficits in motor skill acquisition and retention compared to typically developing peers. They also demonstrate difficulty generalizing learned motor tasks to new contexts [3]. These motor challenges are often compounded by impaired working memory [4], which undermines the effectiveness of learning approaches reliant on conscious cognitive processing. Movement patterns in children with DCD tend to be effortful, irregular, and variable across repeated attempts, and their capacity to detect and learn from errors is reduced [5]. These difficulties often lead to withdrawal from physical activities, increasing risks of social isolation, depression, and lower quality of life [6]. Typically, DCD manifests with impairments in both fine and gross motor skills [7], poor postural control, and decreased muscle strength, particularly in the lower limbs [8].

To enhance motor function, physiotherapists employ both implicit and explicit learning strategies. Explicit motor learning involves conscious, verbalized knowledge acquisition and typically progresses from an initial cognitively demanding phase to a more automatic stage [9]. This early phase places high demands on working memory. In contrast, implicit motor learning allows skill acquisition without conscious awareness or substantial verbal knowledge, bypassing the cognitively effortful stages [10]. Implicit learning is particularly advantageous for children with DCD, who commonly experience working memory deficits [11].

Supporting this, research indicates children with DCD can successfully acquire motor sequences through implicit processes [12]. Given that working memory limitations hamper explicit learning effectiveness, practice methods emphasizing implicit learning are expected to be more beneficial for this population [13]. For instance, Krajenbrink et al. (2023) found that children with DCD can perform dual tasks comparably to peers but expend more mental effort, underscoring the importance of learning strategies that reduce cognitive load [14].

Various implicit learning paradigms have been explored, including reducing verbal instructions [15], dual-task learning [16], errorless learning [17], reduced feedback [18], subliminal learning [19], analogy learning [20,21], and external attentional focus [22]. This study concentrates on analogy learning and external focus of attention, both of which have strong empirical support as implicit learning techniques.

Analogy learning simplifies complex motor patterns into a single, vivid metaphor, providing learners with a meaningful mental image that encapsulates the movement [23]. For example, a coach might instruct a learner to “swing your arm like a pendulum,” reducing the need for detailed biomechanical explanations. External focus of attention involves directing the learner’s attention toward the effects of their movements on the environment (e.g., focusing on the ball’s trajectory) rather than on their own body movements. Both methods minimize cognitive load and have demonstrated superior motor performance under dual-task conditions [20].

Although traditionally studied independently, analogy learning and external attentional focus may operate via overlapping mechanisms. The use of analogies can facilitate sustained external focus by providing clear, goal-directed imagery, potentially enhancing implicit learning efficiency [24]. Empirical studies support the effectiveness of analogy learning in typically developing children; for example, it improved rhythmic movement [25] and rope skipping skills [26], with superior retention and transfer outcomes compared to explicit instruction. Tse et al. (2019) also reported enhanced basketball shooting performance in children with autism following visual analogy instruction, outperforming verbal analogy and explicit methods [27]. Furthermore, Chatzopoulos et al. (2020) found that analogy instruction uniquely improved balance in preschool children, alongside gains in running and jumping [28].

Despite promising results in typical populations, research on analogy learning for children with developmental disorders like DCD is scarce. Moreover, while studies in typically developing children have demonstrated benefits of analogy learning [25,27], not all research has yielded uniformly positive results. Several studies have reported null or mixed findings regarding the superiority of analogy over explicit instruction. For instance, Bobrownicki et al. (2015) found no statistically significant performance differences between analogy and explicit instruction in high jump learning among novices, despite observing more efficient technique in analogy learners [29]. Similarly, Bobrownicki et al. (2019) reported no significant differences in motor control outcomes between analogy and explicit instructional types during acute performance, with both conditions actually reducing accuracy compared to baseline [30]. In a recent study of drawing movements, Berman et al. (2020) found that explicit instruction outperformed analogy instruction on certain kinematic measures (coarticulation), leading the authors to conclude that “every pair of task and analogy is unique, and they must be aligned in order to have an effect” [31]. Even in studies reporting positive outcomes, effects are not always consistent across all measures; for example, Meier et al. (2020) found that while both analogy and explicit instruction improved mental representation structures in junior tennis players, there were no significant performance differences between instructional groups [32]. These inconsistent findings suggest that the efficacy of analogy learning may depend on factors such as task characteristics, the quality of the analogy-task fit, learner skill level, and individual cognitive differences—factors that remain poorly understood, particularly in populations with developmental disorders. Additionally, most analogy learning studies have been conducted with typically developing populations or specific clinical groups (e.g., autism) [27], leaving a critical gap regarding its efficacy for children with DCD, who face unique working memory and motor planning challenges that may differentially impact their response to implicit learning strategies.

In contrast, extensive evidence supports the benefits of external attentional focus in enhancing motor performance. According to the constrained action hypothesis, external focus promotes automaticity, reduces working memory demands, and decreases movement variability by facilitating unconscious motor control [33]. Adults consistently demonstrate better performance and learning under external focus instructions compared to internal focus [34]. Similarly, children aged 8–12 years show improved accuracy and smoother execution with external focus cues [35].

Children with neurodevelopmental disorders also benefit from external focus: those with ADHD [36] and DCD [37] have exhibited superior motor outcomes when instructed to adopt an external attentional focus. However, findings are not universally consistent. Fathi-Khatab et al. (2018) observed that typical children performed better with internal focus, while no differences were found in children with DCD [38]. Similarly, Van Cappellen et al. (2018) reported no performance advantage of external over internal focus in children with DCD, although visuospatial working memory capacity moderated performance [39]. Jarus et al. (2015) found no significant differences in motor learning between attentional focus conditions among children with DCD [40].

These conflicting findings suggest that external focus alone may be insufficient for optimizing implicit motor learning in children with DCD. Several factors may account for these inconsistencies, including differences in task complexity [40], individual variations in visuospatial working memory capacity [39], developmental stage, and the specific wording or salience of attentional focus cues [38]. The heterogeneity in DCD symptom presentation and severity may also contribute to variable responses to attentional focus manipulations. These inconsistencies underscore the need for alternative or complementary implicit learning approaches that may be more robust across diverse task demands and individual differences in this population. Given that analogy learning can facilitate improved movement mechanics without conscious control [24], combining analogy instruction with external attentional focus may create more optimal implicit learning conditions. The metaphorical nature of analogies might also ease the attentional shift from internal to external focus, reducing competition from body-focused cues.

Therefore, this study aims to investigate whether the combined use of analogy instruction and external focus cues produces additive or synergistic benefits in motor skill learning in children with DCD. Using a golf putting task requiring precision and coordination, the research compares combined instruction against single-method and control conditions.

To deepen understanding of motor learning mechanisms, this study also examines participants’ mental representation structures, based on the cognitive action architecture approach. This theory posits that motor actions are controlled by evolving mental representations in long-term memory, which reflect learning-induced cognitive adaptations [41]. Prior work has shown that mental representation structures become more expert-like following practice [32], supporting the notion that changes in mental representation accompany motor skill acquisition. Hence, a secondary objective is to compare the evolution of mental representation structures across implicit (analogy + external focus) and explicit instructional groups in children with DCD. Investigating these cognitive changes provides a more comprehensive view of how motor learning occurs in this population.

Based on the theoretical rationale outlined above, we hypothesize that the combined analogy and external focus instruction may yield greater improvements in motor performance and retention than either method alone or traditional instruction, although the limited empirical base necessitates an exploratory approach. We further hypothesize that performance under dual-task conditions will be more stable in the combined group, reflecting reduced working memory dependence and enhanced implicit learning.

Overall, this study addresses key gaps in the motor learning literature by exploring combined implicit learning strategies in children with developmental and cognitive challenges, assessing both behavioral performance and underlying cognitive representations. The findings aim to inform more effective, evidence-based interventions for motor skill development in children with DCD, enhancing physical education and therapeutic practices.

## Methodology

The present research is a quasi-experimental and applied research conducted as a randomized groups pre-test and post-test design with a control group. This research was conducted in five phases: pre-test, acquisition, retention, transfer, and dual task.

### Participants

A total of 80 children with developmental coordination disorder in the age range (7−9 years) from Tehran city were initially assessed for eligibility. Inclusion criteria required a total score below 62 (≤ 15th percentile) on the MABC-2 test, indicating the presence of movement difficulties.

The required sample size was determined a priori using G*Power (version 3.1.7). An F-test family was selected with the statistical test “ANOVA: Repeated measures, within–between interaction”. The effect size (f = 0.23) was adopted from Liao & Masters (2001) and corresponded to the expected group × time interaction, which was the primary effect of interest [42]. The parameters were set as follows: α = 0.05 (two-tailed), power (1–β) = 0.85, number of groups = 5, number of measurements = 3, assumed correlation among repeated measures = 0.50, and nonsphericity correction ε = 1. The analysis indicated a required total sample size of 60 participants, resulting in 12 participants per group.

Participants were assigned to five groups using a block randomization procedure to ensure equal group sizes: Analogy Instruction, External Attention, Internal Attention, Analogy Instruction + External Attention, and Control. The randomization sequence was generated by an independent researcher using a computer-based random numbers table. Allocation concealment was maintained by the researcher responsible for enrollment, and assessment was blinded to group assignments. No adverse events, dropouts, or protocol deviations occurred. All 60 participants completed the protocol, resulting in 100% retention. Consequently, intention-to-treat and per-protocol analyses are identical.

The randomization sequence was generated using a computer-based random number generator (Microsoft Excel RAND function). Each participant was assigned a sequential identification number (1–60), and group allocation was determined by random assignment to ensure equal distribution across the five conditions. Randomization was performed by a research assistant not involved in outcome assessment after all baseline measurements were completed. Group assignments were recorded in a secure file accessible only to the individual delivering the interventions. Given the nature of the verbal instructional interventions (analogy metaphors vs. explicit technical cues), it was not feasible to blind participants or the instructor delivering the interventions to group allocation. However, to minimize potential bias, outcome assessors measuring putting accuracy used objective, standardized procedures (radial error measured to the nearest millimeter using a calibrated measuring tape), and the assessor conducting mental representation evaluations (SDA-M) was blinded to participants’ group allocation and putting performance results. Additionally, all outcome measures were recorded before participants received any intervention, and the same standardized protocols were followed for all participants regardless of group.

Participants were recruited between [20/04/2025] and [27/07/2025]. All participants had normal or corrected-to-normal vision, were free of neurological or motor disorders, had limited experience with the golf putting task, and provided informed consent, which were among the criteria for entry into the present research. To ensure eligibility, we reviewed the available medical records at the schools for all potential participants. This review confirmed the absence of diagnosed neurological or motor conditions such as ADHD, ASD, or significant visual impairments. Additionally, parents or guardians completed a health screening form that explicitly inquired about any current medication use. No participants were excluded based on comorbid diagnoses or medication at the time of enrollment.

The Movement Assessment Battery for Children Second Edition (MABC-2) was used for screening children with DCD. This tool, in addition to being one of the most commonly used instruments in this field, is an assessment tool that can be easily used in school environments and is a valid and reliable instrument. The validity of the Persian version of the MABC-2 test has been demonstrated in Iranian populations [43]. This test is used to identify and describe disorders in motor performance of children and adolescents aged 3–16 years, which are divided into three age groups: 3–6 years, 7–10 years, and 11–16 years. This tool consists of two functional sections and a checklist. Individuals complete a set of eight fine and gross motor tasks that are divided into three subscales: manual dexterity skills, aiming and catching skills, and balance [44].

In the MABC-2 test, a traffic light system with three colors—green, yellow, and red—has been employed to facilitate and assist in score interpretation, which applies to both the checklist and performance test. In the performance test, based on the relevant norms, any participant who obtains a standard score of 5 (equivalent to the 5th percentile) is considered an individual with significant and meaningful motor impairment and is placed in the red zone. A standard score of 7, equivalent to percentiles between 6 and 15, represents an individual at risk (probability of motor problems) in the yellow zone, while individuals above the 16th percentile, who are unlikely to have motor problems, are placed in the green zone, In this study, a total score of 62 on the MABC-2 scale is regarded as a diagnostic criterion; total MABC-2 score < 62 (bottom 15%) signifies the presence of at least one movement disorder in children [44].

The psychometric properties of the Movement Assessment Battery for Children – Second Edition (MABC-2) have been confirmed in the Iranian population. A validation study involving 503 typically developing children aged 7–10 years supported the original three-factor structure with excellent fit indices. Internal consistency was acceptable (Cronbach’s α = 0.64), and both test–retest (ICC = 0.99) and inter-rater reliability (ICC = 0.86) were high, indicating robust reliability for use in this context [43].

The DCD-Q′07 questionnaire was also used to measure DCD symptoms [45]. This questionnaire consists of 15 items with behavioral descriptions (e.g., “Your child throws a ball in a controlled and accurate manner”). Items are answered on a 5-point Likert scale ranging from “not at all like my child” to “extremely like my child.” According to the child’s age, a total score below 46–57 is considered as “indicative of DCD.” The internal consistency of this questionnaire has been evaluated as excellent (Cronbach’s alpha = 0.94) [46]. Also, the overall validity of the questionnaire within the Iranian population was assessed using Cronbach’s alpha coefficients of .83 and test–retest reliability of .93, respectively [47].

Ethical approval for this study was obtained from the Research Ethics Committee of Damghan University, Iran (Approval Number: IR.DU.REC.1404.002, Date: 2025-04-07). All procedures complied with the Declaration of Helsinki and ethical standards for research involving children with developmental disabilities. Recruitment occurred through specialized motor rehabilitation centers with permission from the center directors. Written informed consent was obtained from parents or legal guardians of all child participants, who were provided with detailed written and verbal information about the study’s purpose, procedures, potential risks, and benefits. Verbal assent was obtained from each child participant following an age-appropriate explanation of the study activities. Families were explicitly informed that participation was voluntary, that they could withdraw at any time without penalty, and that withdrawal would not affect any services their child was receiving. All participant data were anonymized, and confidentiality was strictly maintained throughout the research process.

### Research design

In this study, before beginning practice, participants were instructed and trained on how to grip and raise the hand for striking, how to hold the putter, as well as proper foot positioning and ball placement (feet spread shoulder-width apart with the ball centered between the feet). Participants were required to strike a golf ball in such a manner that it would land in the center of the target. A 70-centimeter Spalding putter was used for shorter children, while a 77.5-centimeter Spalding putter (Spalding, Bowling Green, KY) was used for taller children, along with standard white golf balls (Titleist Pro V1, 4.7 cm in diameter). Strikes were performed on a flat green surface (3 meters wide and 9 meters long) toward a target (10.8 cm) from a distance of 5 meters. A measuring tape was used to measure the distance of the ball from the target hole. In the present research, a pilot study was utilized to determine the appropriate distance [35].

### Test phases

*Pre-test Phase*: In the pre-test, each participant performed 20 attempts from a 5-meter distance from the target. In both the familiarization and pre-test phases, no visual feedback from the target was provided. This was done to reduce the amount of short-term learning that could occur during the pre-test phase.

*Acquisition Phase:* In this phase, participants were assigned to one of five groups based on their pre-test accuracy scores: analogical instruction, external focus, internal focus, analogical instruction-external focus, and control group (12 participants per group). During the acquisition phase, participants practiced golf putting skills over three consecutive sessions, performing 60 trials from a specified distance of 5-meter to the target (three blocks of 20 trials) with a 1-minute rest between each block (participants were asked to sit on a chair during this rest period), totaling 180 trials according to their respective group protocols. Participants in the learning group were asked to act based on a simple biomechanical metaphor and perform the golf putt with a pendulum-like swinging motion [20,21]. In the internal focus group, participants were asked to execute based on instructions provided by the coach and focus on their striking hand during execution. In the external focus group, participants were asked to execute based on instructions provided by the coach and focus on the racket’s movement path and target. Participants in the combined group (analogical instruction + external focus) were asked to simultaneously focus on the pendulum-like golf putt motion while concentrating on the pendulum movement of the racket and the strike target. The control group did not receive analogical instructions or attentional focus instructions but practiced the task with an equal number of attempts as the other groups. Participants received no verbal feedback or error correction but could observe whether their strike hit the target or not. Seventy-two hours after acquisition, participants returned to the laboratory to perform golf putting, and retention and transfer tests were conducted exactly like the pre-test.

*Retention (Immediate and Delayed) and Transfer Phases:* In this phase, participants took part in the immediate retention test immediately after the acquisition phase and were recalled 72 hours later to perform retention and transfer tests [41]. Each participant first performed 2 warm-up trials and then conducted retention tests similar to the pre-test by performing 20 trials from a 5-meter distance. The target in the retention test was positioned at the same distance as the pre-test phase, while the transfer test required participants to perform golf putting 0.5 meter farther from the pre-test/retention target (performing 20 trials 5.5 meters from the starting position) toward the target.

*Dual-Task (Automaticity) Transfer Test:* To assess the degree of automaticity developed through different instructional approaches, participants performed the putting task under dual-task conditions. Participants were instructed to maintain their putting accuracy while simultaneously performing a secondary cognitive task. The secondary task consisted of auditory tone counting: high- and low-pitched tones (2000 Hz and 500 Hz, respectively) were randomly presented through two speakers positioned near the putting area (1 meter behind and to either side of the participant) throughout the trial. Tones were generated using computer software and presented at clearly distinguishable intensities. Tone Presentation Parameters are:

- **Number of tones per trial:** A total of 8–12 tones were presented during each putting trial, with 4–6 high-pitched tones and 4–6 low-pitched tones. The exact number varied randomly across trials to prevent anticipation.
- **Timing and duration:** Tones were presented at random intervals (minimum 2-second separation to ensure discriminability) beginning when the participant initiated their putting motion and continuing until the ball came to rest. Each tone lasted approximately 0.5 seconds. The total duration of tone presentation per trial was approximately 20–30 seconds, corresponding to the time required to complete the putting motion.
- **Synchronization with putting:** Tone presentation began simultaneously with the participant’s backswing initiation (signaled by the experimenter saying “ready, begin”) and continued throughout the putting execution until ball movement ceased. This ensured that participants had to maintain attention to the counting task throughout the entire motor action.

Instructions and Task Priority: Participants received the following standardized instructions before beginning the dual-task test: “In this test, you will put the ball just like before, but you will also hear high and low sounds coming from the speakers. Your job is to do two things at the same time: (1) try to get the ball as close to the hole as possible, just like you’ve been practicing, and (2) count only the high sounds—the ones that sound like ‘beep beep.’ Don’t count the low sounds. At the end of each shot, I will ask you how many high sounds you heard, and you will tell me the number. Both jobs are equally important—try your best on putting AND counting.”

Immediately after each putting trial (after the ball stopped moving), the experimenter asked, “How many high sounds did you count?” The participant verbally reported their count, which was recorded by the experimenter. The actual number of high-pitched tones presented was also recorded for each trial.

Secondary-task performance was quantified by calculating the absolute counting error for each participant: |actual number of high-pitched tones – reported number of high-pitched tones|. Lower error scores indicate better secondary-task performance (more accurate counting). This measure allowed us to verify that all groups engaged equivalently with the secondary task, ensuring that group differences in putting performance under dual-task conditions reflected genuine differences in automaticity rather than differential task prioritization or compliance. Participants were explicitly told to try their best on both tasks and that both putting accuracy and counting accuracy were equally important. This instruction was intended to minimize strategic trade-offs between tasks.

In the dual-task test from the same distance as retention (5 meters), performing 20 trials, two sounds were randomly presented via metronome at different intensities. The absolute counting error per trial was calculated as the absolute difference between the actual and reported number of high tones (|actual high tones – reported high tones|). The mean of this error across 20 trials was then computed for each participant. This measure was used to verify equivalent secondary-task engagement across the different groups [20,42]. Table 1 shows the practice protocol for different groups at various test phases.

**Table 1:** Design of the study including five tests and an acquisition phase of three days.

Group	Pre-test	Acquisition (3 days)	Retention test (72 hour)	Transfer test (72 hour)	Transfer test (Dual task)
Analogy (n = 12)	SDA-M* 20 trials (5 m)	**20 trials (5 m)** **20 trials (5 m)** **20 trials (5 m)**	SDA-M 20 trials (5 m)	**20 trials (5.5 m)**	SDA-M 20 trials (5.5 m)
Analogy-External (n = 12)	SDA-M 20 trials (5 m)	**20 trials (5 m)** **20 trials (5 m)** **20 trials (5 m)**	SDA-M 20 trials (5 m)	**20 trials (5.5 m)**	SDA-M 20 trials (5.5 m)
Internal (n = 12)	SDA-M 20 trials (5.5 m)	**20 trials (5 m)** **20 trials (5 m)** **20 trials (5 m)**	SDA-M 20 trials (5 m)	**20 trials (5.5 m)**	SDA-M 20 trials (5.5 m)
Internal (n = 12)	SDA-M 20 trials (5.5 m)	**20 trials (5 m)** **20 trials (5 m)** **20 trials (5 m)**	SDA-M 20 trials (5 m)	**20 trials (5.5 m)**	SDA-M 20 trials (5.5 m)
Control (n = 12)	SDA-M 20 trials (5.5 m)	No practice	SDA-M 20 trials (5 m)	**20 trials (5.5 m)**	SDA-M 20 trials (5.5 m)

*Note: SDA-M: Structural dimensional analysis of mental representation

Assessment of Participant Accuracy: To measure the performance of each strike, the coordinates of each strike’s landing position were recorded, and the final stopping distance from the ball’s edge to the inner edge of the target was measured on both horizontal (x) and vertical (y) axes using a measuring tape. Accuracy was measured using Mean Radial Error (MRE), where MRE represents the mean radial error. RE is the radial error (mean radial error was calculated as the average distance of each result from the target center on both horizontal (x) and vertical (Y) axes in centimeters. M represents the number of attempts and I represents the specific attempt. The MRE equation is presented as Equation 1 [48].

Equation 1:


$$\begin{array}{c} MRE=\frac{\text{1}}{\text{2}\text{0}\;}\sum_{i}^{\text{2}\text{0}}REi \end{array}$$

$$\begin{array}{c} RE=\sqrt{X^{\text{2}}+Y^{\text{2}}} \end{array}$$

$$\begin{array}{c} {RE=\left(x^{\text{2}}+Y^{\text{2}}\right)}^{\text{1}/\text{2}} \end{array}$$

### Primary and secondary outcomes

**Co-Primary Outcomes:** This study specified two co-primary outcome measures, each addressing a distinct aspect of motor learning:

1. **Delayed Retention MRE** (72 hours post-acquisition): Assesses long-term learning and skill retention, providing evidence that learning persists beyond the training context.
2. **Dual-Task Transfer MRE:** Assesses movement automaticity and the degree to which motor control is resistant to disruption by concurrent cognitive demands, directly testing predictions from implicit learning theory.

Both outcomes were considered equally important for evaluating the effectiveness of different instructional approaches. To control for multiple primary comparisons, we used a Bonferroni-corrected alpha level for hypothesis testing of group effects on these two primary outcomes.

#### Instructional scripts and delivery procedures.

To ensure consistency of instruction delivery across all participants within each group, standardized verbal scripts were developed and used by the instructor. The same instructor delivered all interventions to minimize inter-instructor variability. Below are the exact instructional scripts provided to participants in each group:

1. **Analogy Instruction Group:** Before each 10 trials, participants received the following instruction:

*“I want you to imagine that your putting stroke is like a pendulum on a grandfather clock—it swings smoothly back and forth in a straight line. Let your arms and putter swing naturally like the pendulum, keeping a smooth, steady rhythm. Don’t think about the details of your movement; just let it swing like a pendulum. Remember: swing like a pendulum.”*

No additional technical instructions were provided.

2. **External Focus of Attention Group:** Before each 10 trials, participants received the following instruction:

*“Focus your attention on the path of the putter head as it moves toward the ball and follows through toward the target. Watch the line the putter makes as it travels. Keep your eyes on the putter path, not on your hands or body. Think about: the path of the putter.”*

3. **Internal Focus of Attention Group:** Before each 10 trials, participants received the following instruction:]

*“Focus your attention on the movement of your hands and arms as you strike the ball. Pay attention to how your striking hand moves back and then forward. Think about the motion of your hands throughout the putting stroke. Remember to focus on: your hand movements.”*

4. **Combined Group (Analogy + External Focus):** Before each 10 trials, participants received the following instruction:

*“Imagine you’re putting stroke is like a pendulum—swinging smoothly back and forth. While you do this, focus your attention on the path of the putter head as it moves toward the ball and the target. Let it swing like a pendulum, and watch the putter path. Remember: pendulum swing, watch the putter path.”*

5. **Control Group:** Before each 10 trials, participants received only basic task instructions:

*“Try to get the ball into the hole. Do your best on each shot.”*

No specific attentional focus or technical instructions were provided. Participants received equivalent practice time, number of trials, encouragement, and experimenter contact as all other groups, but without attentional or technical guidance.

#### Fidelity procedures.

To ensure instructional fidelity and consistency across participants:

1. **Standardized scripts:** All verbal instructions were scripted in advance and read verbatim by the instructor at the designated times (before each 10 trials).
2. **Single instructor:** The same trained instructor delivered all interventions to all participants to eliminate inter-instructor variability.
3. **Instructor training:** The instructor was a graduate student trained in motor learning who practiced delivering the scripts until standardization was achieved.
4. **Fidelity monitoring:** Choose the option that matches what you actually did:
  - A subset of sessions (20% randomly selected]) were audio/video recorded
  - Recordings were reviewed by an independent researcher using a checklist to verify: (a) correct script delivery, (b) no additional technical cues provided, (c) equivalent encouragement across groups
  - Fidelity was > 95% across all monitored sessions
5. **Prohibition of additional feedback:** Instructors were explicitly trained to provide ONLY the scripted instructions and general encouragement (e.g., “good effort,” “nice try”). No additional technical feedback, error correction, or elaboration on instructions was permitted. This ensured that groups differed only in the intended instructional manipulation (analogy vs. explicit cues, external vs. internal focus) and not in the amount or type of ancillary coaching.

A standardized protocol was implemented to ensure equivalent contact and attention across all groups, including the control. Specifically, all participants received identical practice time and trial counts, constant experimenter presence and supervision, general encouragement (e.g., “good effort,” “you’re doing well”), and a uniform session structure and duration. The sole manipulated variable was the content of the attentional or instructional cues provided prior to practice blocks. This design ensured that any observed group differences could not be attributed to differential experimenter attention, contact time, or expectancy effects, but were instead a result of the specific instructional approach being tested.

### Structural dimensional analysis of mental representation

The participants’ mental representations were analyzed in the pre-test, retention, and transfer testing phases. In the pre-test phase, the initial level of mental representation structure was analyzed with SDA-M software. Mental representations of golf putting were assessed using 16 Basic Action Concepts (BACs) previously validated and identified through objective psychometric methods by Frank et al. (2013) [41]. These BACs represent the fundamental movement components of the golf putting skill, organized by functional phases of the putting stroke [41]. The 16 basic action concepts of golf putting used in this study were *preparing Phase*: (1) shoulders parallel to the target line, (2) align clubface square to the target line, (3) grip check, (4) look to the hole, *Backswing Phase*: (5) rotate shoulders away from the ball, (6) keep arms-shoulder triangle, (7) smooth transition, *Forward Swing and Contact Phase*: (8) rotate shoulders toward the ball, (9) accelerate club, (10) impact with the ball, (11) clubface square to target line at impact, *Follow-Through Phase*: (12) follow-through, (13) rotate shoulders through the ball, (14) decelerate club, (15) direct clubhead to planned position, (16) look to the outcome. Each BAC was presented to participants as a short verbal phrase. The use of these previously validated BACs ensures comparability with prior SDA-M research on golf putting and provides confidence in the relevance and comprehensiveness of the action concepts assessed. During the pre-test phase, participants were introduced a list of 16 basic action concepts of golf putting in random order, to assess the level of mental representations. The researcher explained the meaning of each basic action concept to the participants.

To analyze learners’ mental representations, the 16 basic action concepts formed the basis of participants’ responses and subsequent analyses [49]. The instructions on the monitor were explained to participants and after the explanation; the participants performed the separation task to determine the structure of their initial mental representation. The separation task that was performed by SDA-M software was the one concept that was permanently displayed as an anchor concept while the other action concepts were randomly displayed; participants were asked to answer whether any of the basic action concepts were related to the anchor concept? After displaying a specific list of action concepts, the next concept was selected as the anchor concept to compare with other concepts. This process was continued and completed until all action concepts were displayed as the anchor concept. We compared the mental structure of the novice participants with the reference structure the mental structure of ten expert golfers who had more than ten years of experience in golf skills.

### Data analyses

**Primary Analyses:** Two primary hypothesis tests were conducted: 1**.** Group differences in delayed retention MRE (one-way ANOVA) 2**.** Group differences in dual-task transfer MRE (one-way ANOVA)**.** To control family-wise Type I error rate across these two primary comparisons, a Bonferroni-corrected alpha was applied to each test.

A data normality and homogeneity of variances were assessed and confirmed by the Shapiro-Wilk tests and the Levene test, respectively (ps > .05). The experimental design of this study consisted of six phases (pre, acquisition, immediate and delayed retention, transfer and dual task tests) for four independent groups (Analogy, Internal, External, Analogy and External Groups).

For the acquisition phase, a mixed design with 5 (Groups: Analogy, Internal, External, Analogy-External, and Control) × 3 (Acquisition Block 1, 2, 3) ANOVA with repeated measures on the last factor was performed with performance score as the dependent variable. The retention phases employed a 5 (groups: Analogy, Internal, External, Analogy External, and Control) × 2 (phases: Pretest, Delayed Retention) ANOVA with repeated measures on the last factor. Furthermore, separate one-way ANOVA was used to analyses the dual-task and transfer tests. All post hoc analyses, Bonferroni’s comparison was used to follow up on any significant effects. We also calculated the effect size, as partial ηp2, where the thresholds for describing the effect sizes as small, moderate, and large were considered as .01 (small), .06 (medium), and .14 (large), respectively [50]. Within-subject effect sizes were calculated using the repeated-measures version of Cohen’s d, which accounts for the correlation between time points when measuring differences between group means [51]. The alpha was set at p = 0.05.

#### Mental representation structures.

We analyzed mental representation structures by calculating mean group dendrograms via cluster analyses [52]. The level of significance for the cluster analysis was set at 5%, which corresponds to a critical value of d_erit_ = 3.41. This critical value (derit) was shown as a horizontal line on the horizontal axis. Relationships between basic action concepts (BACs) above this value are considered statistically unrelated or insignificant. The lower the value of a link between two items, the shorter the distance was between the related BACs in long-term memory. To compare the differences between the cluster solutions, we performed analyses of invariance. In the final step, the invariance of the cluster solutions was determined by measuring: when λ < .68, the two clusters were significantly different while the two clusters were invariant when λ ≥ .68 [53]. Structural similarity between each participant’s mental representation (dendrogram) at each timepoint and the aggregated expert reference structure was quantified using the Adjusted Rand Index [54]. The ARI ranges from −1 to +1, where +1 indicates perfect agreement between two cluster solutions, 0 indicates agreement expected by chance, and negative values indicate less agreement than expected by chance.; thus, higher ARI values reflect greater structural similarity to the expert reference and a shifting toward a more functional structure of action knowledge. To determine the statistical significance of this similarity, each participant’s ARI value at each timepoint was tested against the null hypothesis of chance-level clustering using Monte Carlo permutation testing (10,000 iterations, α = .05), where a significant ARI indicates that the observed structural similarity exceeds random clustering and provides evidence of meaningful cognitive organization. For group-level analysis, mean ARI values were calculated for each group at each timepoint and compared using mixed-design ANOVA, with significant increases in ARI over time interpreted as approaching an expert-like organization of mental representation structures through practice.

A reference expert mental representation structure was established by assessing 10 skilled golfers who served as the benchmark for expert-level cognitive organization of golf putting knowledge. Expert participants were recruited from national golf clubs/associations in Tehran, Iran and met stringent criteria for expertise. The 10 expert golfers (mean age = 28.4 years, *SD* = 3.2; were defined as experts based on the following criteria: 1. **National-level competitive participation:** Active participation in golf competitions at the national level 2. **Deliberate practice volume:** Accumulation of 9,000–10,000 hours of purposeful and deliberate practice in golf skills, consistent with established criteria for expert performance [55] 3. **Competitive experience:** An average of 8–10 years of competitive golf experience and 4. **Systematic coaching:** All skilled participants had received ongoing, systematic coaching from nationally certified golf instructors throughout their competitive careers, with regular training sessions over a minimum of 5 years

The expert sample demonstrated performance characteristics consistent with skilled golf putting as described in the motor control literature. As established by Delay et al. (1997) [56], expert golfers optimize force at the point of contact between the putter head and ball by adjusting backswing amplitude based on distance to the hole while maintaining relatively constant movement velocity across various distances. Expert golfers control the putting stroke by determining downswing amplitude prior to movement initiation, based on spatial parameters such as distance to the hole, green speed, and slope [57–59]. These technical-biomechanical characteristics reflect the sophisticated motor control and cognitive organization that distinguish expert from novice performance.

#### Creation of aggregated expert reference structure.

To create an aggregated expert reference structure, each of the ten experts independently completed the SDA-M split procedure, generating individual mental representation dendrograms. After constructing individual distance matrices and dendrograms via hierarchical cluster analysis, visual and quantitative evaluation revealed high inter-expert consistency in cluster composition, confirming a shared functional organization of golf putting knowledge. To derive a single normative structure, the ten individual expert distance matrices were averaged element-wise to create a pooled matrix, and hierarchical cluster analysis was applied to this matrix to generate the final aggregated expert reference dendrogram. This structure, representing the consolidated expert cognitive organization of the skill, served as the standard against which novice participants’ mental representations were compared at each assessment timepoint (pretest, post-acquisition, delayed retention). The individual expert dendrograms are available upon request, but the aggregated structure is presented as it adequately represents expert-level organization for the study’s comparative analyses.

## Results

Table 2 presents the descriptive statistics for participant demographics and baseline scores across the groups, with values expressed as mean ± standard deviation (M ± SD).

**Table 2:** Participant characteristics of the groups, including means (standard deviations) and scores on the Movement ABC-2 (MABC-2).

*Group*	*AE*	*E*	*A*	*I*	*CON*
	*M± SD*	*M± SD*	*M± SD*	*M± SD*	*M± SD*
*Age*	*8.04 ± 0.47*	*8.03 ± 0.68*	*7.8 ± 4.8*	*7.87 ± 0.74*	*7.79 ± 0.54*
*MABC-2 total score*	*59.58 ± 3.4*	*57.58 ± 1.8*	*59.08 ± 3.2*	*56.08 ± 2.53*	*58.5 ± 3.34*
*MABC-2 total score in percentiles*	*2.4 ± 3.1*	*2.24 ± 3.2*	*2.27 ± 2.8*	*3.4 ± 2.9*	*2.7 ± 3.2*
*DCD-Q′ 07 total score*	*28.65 ± 6.3*	*25.8 ± 4.8*	*26.3 ± 4.8*	*25.09 ± 6.85*	*28.75 ± 7.3*

Note. MABC-2, Movement Assessment Battery for Children 2; DCD-Q′ 07; developmental coordination disorder questionnaire, AE: Analogy-External group, A: Analogy group, E: External group, I: Internal group, & Con: Control group.

### Acquisition phase

#### Statistical model and corrections.

Due to violation of the sphericity assumption (Mauchly’s test of sphericity, p < .05), the Greenhouse-Geisser correction was applied to adjust the degrees of freedom for the within-subjects effects. All post-hoc pairwise comparisons were conducted using Bonferroni correction for multiple comparisons, as implemented in SPSS Statistics (Version 22). For the Group main effect, 10 pairwise comparisons were conducted; for the Acquisition Phase main effect, 3 pairwise comparisons were conducted. All reported p-values for pairwise comparisons are Bonferroni-adjusted values and are evaluated against the conventional alpha level of .05.

#### Main ANOVA results.

The 5 (Group) × 3 (Blocks) mixed factorial ANOVA revealed a significant main effect for Acquisition Phase (F(1.936, 106.456) = 3.967, p = .023, η²p = 0.067) and Group (F(4, 55) = 46.194, p < .001, η²p = 0.771), but did not indicate a significant interaction effect (F(7.742, 106.456) = 1.179, p = .319, η²p = 0.079) (Table 3). A decrease in mean radial error (MRE) was observed across the acquisition session (Blocks 1–3), indicating improved putting accuracy.

**Table 3:** Results of mixed-model ANOVA in phases the acquisition sessions, retention, transfer and dual task.

*Sources*	*Sum of squares*	*Df*	*Mean* *squares*	*F*	*P Value*	*Eta squares* *ƞ2*
***Acquisition***						
*Session*	*141.972*	*1.936, 106.456*	*73.348*	*3.967*	*0.023*	*0.067*
*Group*	*4181.24*	*4, 55*	*1045.31*	*46.194*	*0.001*	*0.771*
*Session × Group*	*168.807*	*7.742, 106.456*	*21.803*	*1.179*	*0.319*	*0.079*
***Retention***						
*Phase*	*7363.91*	*2, 110*	*3681.95*	*345.21*	*0.001*	*0.863*
*Group*	*1647.81*	*4, 55*	*411.79*	*17.48*	*0.001*	*0.56*
*Phase× Group*	*759.43*	*8, 110*	*94.92*	*8.9*	*0.001*	*0.393*
***Transfer***						
*Between Groups*	*998.58*	*4*	*249.64*	*26.17*	*0.001*	*0.656*
*Within Groups*	*524.59*	*55*	*9.538*			
*Total*	*1523.18*	*59*				
*Dual task*						
*Between Groups*	*1151.11*	*4*	*287.77*	*23.54*	*0.006*	*0.631*
*Within Groups*	*672.38*	*55*	*12.225*			
*Total*	*1823.5*	*59*				

#### Post-Hoc pairwise comparisons.

Bonferroni-corrected pairwise comparisons (3 comparisons total) revealed a significant difference between the first and third acquisition blocks (p = .034, d = 0.595, 95% CI = [0.125, 4.174]), indicating overall improvement in performance across practice. No other pairwise comparisons between acquisition blocks reached significance after Bonferroni correction. Bonferroni-corrected pairwise comparisons (10 comparisons total) revealed that all experimental groups (External Focus, Internal Focus, Analogy, and Combined) demonstrated significantly better performance than the Control group (p < .01 for all comparisons). Both implicit learning groups, External Focus (p = .029, d = 0.838, 95% CI = [−6.77, −0.22]) and Combined (p = .003, d = 0.967, 95% CI = [−7.6, −1.05]) demonstrated significantly superior performance compared to the Internal Focus group. Effect sizes were calculated using Cohen’s d = (M₁ – M₂) / SD_pooled, where SD_pooled = √[((n₁ − 1)SD₁² + (n₂ − 1)SD₂²) / (n₁ + n₂ − 2)].

#### Retention phase.

The analysis comparing pre-test to retention tests mixed-design ANOVA results revealed a significant Group × Phase interaction (F(8, 110) = 8.9, p = .001, ηp² = .393), A main effects of Phase, (F(8, 110) = 8.9, p = .001, ηp² = .393) and group (F(8, 110) = 8.9, p = .001, ηp² = .393) were also observed. Because the interaction effect was statistically significant, follow-up analyses were conducted to decompose the interaction. One-way ANOVAs were performed to compare the five groups at each testing phase (pretest, immediate retention, delayed retention). Additionally, paired-samples t-tests were conducted to compare performance between testing phases within each group (pretest vs. immediate retention; pretest vs. delayed retention).

#### Between-group comparisons at each testing phase.

*Pretest:* The one-way ANOVA revealed no significant differences among the five experimental groups at pretest (F(4, 55) = 0.396, p = 0.811, η²p = 0.028), confirming successful random assignment and baseline equivalence.

*Immediate Retention:* The one-way ANOVA revealed significant group differences (F(4, 55) = 15.943, p = 0.001, η²p = 0.537). Bonferroni-corrected post-hoc comparisons indicated that all groups except the Internal Focus group performed significantly better than the Control group (p < 0.05 for all comparisons). Furthermore, all three implicit learning groups, Analogy (p = 0.028, d_Cohen_ = 1.605, 95% CI = [−11.12, −0.37]), External Focus (p = 0.003, d_Cohen_ = 1.463, 95% CI = [−12.537, −1.79]), and Combined (p = 0.001, d_Cohen_ = 2.029, 95% CI = [−13.537, −2.79]), demonstrated significantly superior performance compared to the Internal Focus group.

*Delayed Retention:* The one-way ANOVA revealed significant group differences (F(4, 55) = 18.535, p = 0.001, η²p = 0.574). Bonferroni-corrected post-hoc comparisons showed that all experimental groups, including the Internal Focus group, performed significantly better than the Control group (p < 0.05 for all comparisons). However, only the External Focus group (p = 0.008, d_Cohen_ = 1.216, 95% CI = [−10.157, −0.955]) and Combined group (p = 0.003, d_Cohen_ = 1.607, 95% CI = [−10.62, −1.41]) demonstrated significantly superior performance compared to the Internal Focus group. The Analogy group did not differ significantly from the Internal Focus group (p = 0.067).

#### Within-group comparisons at each groups.

The results of the dependent t-tests also showed that there was a significant difference in all research groups between the pre-test and the immediate retention test and between the pre-test and the delayed retention test. The Table 4 shows the significance level and the effect size of the pairwise comparisons.

**Table 4:** *P*-value and effect size (d Cohen) in comparison between pre-test and immediate and delayed retention tests in research groups.

		Analogy	Internal	External	Combined	Control
Pre-test Immediate Retention	P	0.001	0.001	0.001	0.001	0.01
	d_Cohen_	4.79	1.978	3.8	4.362	0.951
Pre-test Delayed Retention	P	0.001	0.001	0.001	0.001	0.001
	d_Cohen_	4.355	2.257	4.151	4.4	1.505

#### Transfer at a longer distance.

Golf-putting accuracy in transfer was analyzed separately using one-way analysis of variance (Tables 3). The one-way ANOVA showed that a significant main effect groups in the transfer phase (F (4, 55) = 26.174, p = .001, ƞp2 = 0.656). The post-hoc test results showed the on the transfer test from a novel distance (5.5 m), the A, AE and E groups, (M = 52.064, SD = 2.97; M = 49.48, SD = 2.51; M = 52.15, SD = 3) had significantly higher putting accuracy scores (smaller MRE values) compared to the I and CON groups (M = 55.88, SD = 3.51; M = 61.42, SD = 2.6), respectively (ps < 0.001). No significant difference was observed in the transfer test among the, A, AE and E groups.

#### Transfer under dual-task condition.

To verify that group differences in putting performance under dual-task conditions reflected genuine automaticity rather than differential task prioritization, we analyzed secondary-task (tone counting) accuracy across groups. A one-way ANOVA comparing absolute counting errors revealed no significant differences among the five experimental groups, *F*(4, 55) = 0.097, *p* = 0.983, η²p = 0.007. Mean absolute counting errors were: Analogy (*M* = 2.37, *SD* = 0.937), External Focus (*M* = 2.08, *SD* = 0.996), Combined (*M* = 2.16, *SD* = 1.05), Internal Focus (*M* = 2.33, *SD* = 0.985), and Control (*M* = 2.19, *SD* = 1.03). The absence of significant group differences in secondary-task performance indicates that all groups engaged comparably with the auditory counting task, ruling out the possibility that superior putting performance in the implicit learning groups resulted from strategically deprioritizing the secondary task. This finding supports the interpretation that group differences in dual-task putting performance genuinely reflect differences in movement automaticity, with the implicit learning groups (analogy, external focus, combined) demonstrating more automatic motor control that was less disrupted by concurrent cognitive demands.

**Fig 1:**
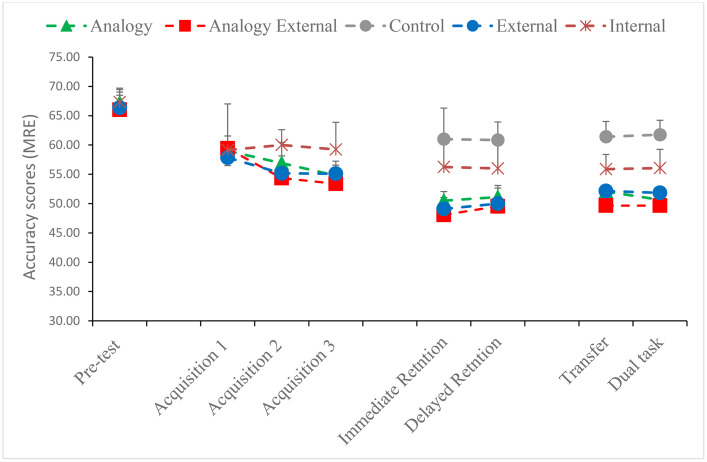
Mean radial error as a function of block and attentional condition. Error bars represent 95% confidence intervals.

The post-hoc test results showed the on the transfer under dual-task test, the A, AE and E groups, (M = 50.064, SD = 2.89; M = 49.67, SD = 3.1; M = 51.82, SD = 2.65) had higher accuracy scores (smaller MRE values) than the I and CON groups (M = 56.08, SD = 5.1; M = 61.76, SD = 2.46), respectively. No significant difference was observed in the transfer test among the three, A, AE and E groups. Fig 1 displays the mean radial error and 95% confidence intervals across different test phases for the four experimental groups.

### Mental representation structure

We used SDA-M software version 1.4.5 [53,60] to analyze the mental representation structure; to do this the mental representation structure of the groups in the pre-test was first examined. For all analyzes, the alpha level was α = .05 with a critical value of = 3.41 derit. related basic action concepts formed into clusters below the critical value and led to a grouping of basic action concepts, cluster structures between the skilled and less skilled groups were considered significantly different at λ < 0.68 (λkrit = 0.68) [49].

#### Pre-test phase.

**Fig 2:**
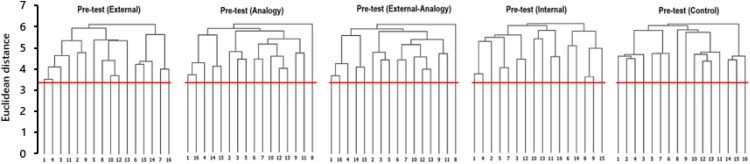
Dendrograms of groups constructed from hierarchical cluster analysis of basic action concepts in golf putting tasks in the pretest phase.

As seen in Fig 2, cluster analysis revealed no significant clustering in the mean EL, EF, groups dendrograms of each group at pre-test (with critical value dcrit = 3.41). Interconnected concepts that are underlined in red are considered meaningful. In fact, there was no significant cluster in the structure of the mental representation of groups. Moreover, the adjusted Rand Index (ARI) indicated that the External-Analogy, External, and Analogy groups became more similar to the mental representation structure of skilled golfers as a result of practice.

The ARI from pretest to retention‐test and pretest to transfer were as follows: External (from 0 0.06 to 0.39, from 0.06 to 0.54), Analogy (from 0.10 to 0.45, from 0.10 to 0.49), and External-Analogy(from 0 [zero] to 0.48, from 0 [zero] to 0.71), Internal (from 0.06 to 0.19, from 0.06 to 0.21), Control (from 0.04 to 0.06, from 0.04 to 0.08).

#### Retention phase.

**Fig 3:**
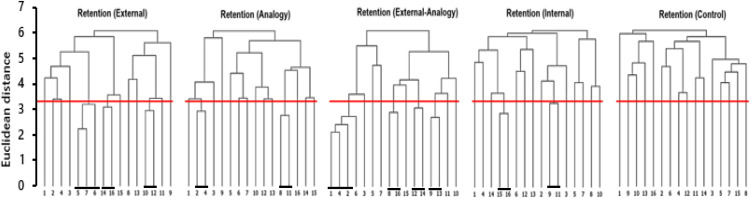
Dendrograms of groups constructed from hierarchical cluster analysis of basic action concepts in golf putting tasks in the retention phase. The horizontal line indicates the critical Euclidean distance. The critical value of the Euclidean distance (dcrit) was 3.40 for an α level of 5%. Clusters below this line indicate statistically significant, while clusters above this line indicate statistically insignificant.

As the results of Fig 3 in the retention phase show, after the acquisition phase, the mean dendrograms of the groups changed and improved significantly so that the structure of mental representation of the AE group consists of four functional clusters related to different phases of movement.

Invariance analysis comparing the structure of mental representations among groups during the pre-test, retention, and transfer tests revealed significant differences in the mental representation structure of the AE, A, and E groups between the pre-test and retention test (λ < 0.68) (Table 5). Furthermore, the analysis aimed to determine whether there were statistically significant differences between the test phases. The structures of mental representation of five groups in both the pre-test and retention test, as well as the pre-test and transfer test, were significantly different, indicating notable improvements in their mental representation structures (λ < 0.68). Specifically, the results were as follows: External (λ = 0.32, p < 0.05; λ = 0.37, p < 0.05), Analogy (λ = 0.34, p < 0.05; λ = 0.43, p < 0.05), External -Analogy (λ = 0.44, p < 0.05; λ = 0.47, p < 0.05), Internal (λ = 0.28, p < 0.05; λ = 0.27, p < 0.05), and Control (λ > 0.69, p > 0.05; λ > 0.71, p > 0.05).

**Table 5:** Adjusted Rand Index (ARI) values from pre-test (first value) to retention and transfer tests (second value), with cluster solutions classified as variant (ARI < 0.68) or invariant (ARI ≥ 0.68).

Group	Pre-test to Retention	Pre-test to Transfer
Analogy (n = 12)	λ =0.34 ARI= = 0.10 to 0.45	λ =0.43 ARI= = 0.10 to 0.49
External (n = 12)	λ =0.32 ARI= from 0.06 to 0.39	λ = 0.37 ARI= = 0.06 to 0.54
External -Analogy (n = 12)	λ =0.44\ ARI= = 0 to 0.48,	λ =0.47 ARI= = 0 to 0.71
Internal (n = 12)	λ =0.28 ARI= = 0.06 to 0.19,	λ =0.27 ARI= = 0.06 to 0.21
Control (n = 12)	λ = 0.69 ARI= = 0.04 to 0.06	λ = 0.71 ARI= = 0.04 to 0.08

In the retention-test, the results of the hierarchical cluster analysis show connections that fall below the critical value (indicated by the horizontal red line) which are statistically considered as connections. In contrast, connections above this line are regarded as distinct clusters. As illustrated, three distinct functional clusters have formed within the external group, corresponding to different phases of the action: the backswing phase, the Attenuation phase, and the impact phase (i.e., BAC 5,7,14,16,10,12). BAC,2,4,8,11). Also, two distinct functional clusters have formed within the external analogy group, corresponding to different phases of the action the Preparation phase, the backswing phase, the forward swing phase and the impact phase and the Attenuation phase(i.e., BAC,1,4,2,6,8,16,12,14,9,13). two distinct functional clusters have formed within the internal group, the forward swing phase and the impact phase and the Attenuation phase (i.e., BAC,15, 16, 10,10). However, no significant clusters are observed within the control group.

**Fig 4:**
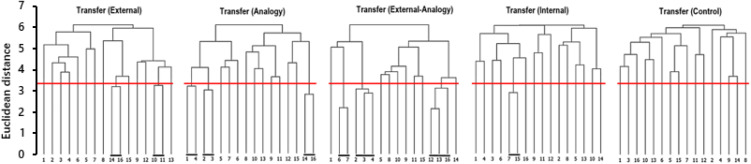
Dendrograms of groups constructed from hierarchical cluster analysis of basic action concepts in golf putting tasks in the transfer phase. The horizontal line indicates the critical Euclidean distance. The critical value of the Euclidean distance (dcrit) was 3.40 for an α level of 5%. Clusters below this line indicate statistically significant, while clusters above this line indicate statistically insignificant.

In the transfer under dual-task, the results of the hierarchical cluster analysis show connections that fall below the critical value (indicated by the horizontal red line) which are statistically considered as connections (Fig 4). In contrast, connections above this line are regarded as distinct clusters. As illustrated, two distinct functional clusters have formed within the external group, corresponding to different phases of the action: the impact phase, the Attenuation phase, (i.e., BAC 14,16,10,11). Also, three distinct functional clusters have formed the analogy group, corresponding to different phases of the action: The Preparation phase, the Attenuation phase (i.e., BAC,1,4,2,3,14,16). Also, three distinct functional clusters have formed within the external analogy group, corresponding to different phases of the action the Preparation phase, the backswing phase, the impact phase and the Attenuation phase (i.e., BAC,6,7,2,3,12,13). one distinct functional cluster have formed within the internal group, the forward swing phase and the impact phase and the Attenuation phase (i.e., BAC 7,15). However, no significant clusters are observed within the control group.

## Discussion

The present study examined the effects of analogical learning instructions and attentional focus on motor acquisition and learning in children with DCD. The results showed that the external focus, analogical, and combined (external focus-analogical) groups, but not the internal focus group, experienced significant improvement during the acquisition phases, and there was a significant difference between the combined group (external-analogical) and the internal focus group in the acquisition test (acquisition 2 and 3). The results also showed that significant differences were observed between the three groups (external focus, analogical, and combined) and the internal focus and control groups in both retention tests (immediate and delayed) and transfer tests.

Before discussing the implications of these findings, several important considerations regarding generalizability should be noted. First, our participants were children aged 7–9 years with DCD recruited from specialized centers in Tehran, Iran. Findings may not generalize to other age groups, cultural contexts, or typically developing children. Second, the golf putting task, while providing experimental control, is a sport-specific skill rather than a functional daily living activity. Whether these instructional approaches similarly benefit the everyday motor challenges faced by children with DCD (e.g., handwriting, dressing, ball skills) remains to be established. These limitations should be kept in mind when interpreting the following discussion.

The results of this study demonstrated that practice with all three methods—external focus, analogical, and combined—all of which are considered implicit practice methods, led to better results in both performance and learning compared to internal attentional focus, which represents explicit practice models. These results are consistent with findings from a number of studies [37,39,40]. Van Abswoude et al. (2021) presented evidence from 25 studies in a systematic review that examined the effects of explicit and implicit motor learning methods in typically developing children aged 4–12 years and children with developmental disorders [61]. Among the reviewed studies, four studies used errorless learning, three studies used analogical learning, and 18 studies used external focus instructions as implicit learning methods. They found that implicit learning methods were more effective than explicit learning methods in improving children’s motor performance.

The results of this study are specifically consistent with research demonstrating that using analogical methods compared to explicit learning methods leads to better performance and learning [26–28]. Research indicates that the analogical method uses less working memory, leaving space for additional cognitive load and allowing individuals to retain skills over a longer period. Liao and Masters (2001) proposed the hypothesis that analogy facilitates learning a motor concept/movement because it chunks or segments much of the information that constitutes the learnable skill structure in a way that requires less conscious processing [42]. The use of analogies for learning new motor skills has been shown to improve learning in various environments and tasks, such as basketball shooting or even performing a high jump [62]. A study comparing the effects of analogical learning on children’s running, sprinting, balance, and long jumps showed positive results in favor of analogical learning [28]. Results showed that the analogical learning group’s balance performance improved significantly, while the non-analogical group showed no significant improvement [28]. In another study, the effectiveness of analogical instruction in acquiring complex motor skills and performance under pressure was examined using a modified (seated) basketball shooting task [21]. Both analogical and non-analogical groups performed well during the learning phase. However, the non-analogical group reduced performance during the transfer test under pressure, while the analogical group performed without effect [20]. It is suggested that when skills are learned through analogical learning, children maintain stable performance under pressure conditions, when facing situations requiring decision-making, and in dual-task conditions [62,63].

Additionally, in the domain of attentional focus, the results of the present study are consistent with research demonstrating the superiority of external attentional focus over internal focus in children [35,37,64]. Based on theoretical accounts [33] and indirect behavioral evidence from dual-task performance, external focus may reduce working memory demands, potentially making it suitable for individuals with reduced working memory capacity. However, direct measurement of working memory load during learning would be needed to confirm this mechanism. Furthermore, according to the constrained action hypothesis [33], external attentional focus—that is, focusing on the outcome of movement rather than the movement itself—increases the automaticity of movement execution [65]. This increased movement automaticity reduces working memory demands because unconscious control processes primarily regulate movement control. Therefore, external focus is supposed to promote implicit motor learning [10]. In contrast, if attentional focus is directed toward the learner’s body movements—that is, internal attentional focus—explicit motor learning is supposed to be facilitated. Internal attentional focus suggests working memory involvement because it creates a conscious control state that interferes with the natural automatic control processes of movement execution [33].

One of the interesting findings of the present study is the better performance of the combined group (analogical-external focus) in the second and third acquisition sessions compared to the internal focus group, while neither the separate external focus nor analogical groups showed significant differences with the internal focus group during the acquisition phase. The superiority of the combined group (analogical-external focus) compared to the control group in acquisition sessions can be explained based on two approaches:

First, we hypothesize that simultaneous use of these two models may result in greater reduction of working memory load compared to separate conditions, though this mechanism was not directly measured in the present study. The superior dual-task performance in the combined group is consistent with this interpretation, but direct assessment of working memory demands would be needed to test this hypothesis. It seems that reducing working memory load will ultimately lead to greater self-organization of the motor system and execution with increased automaticity [33,66]. This is evidenced by the automaticity test results of the present study, which showed that the combined group’s performance was significantly better than the internal focus group.

Second, simultaneous use of the external focus model and analogical learning creates positive synergy in directing the learner’s attention to the environment. Although in external focus individuals are asked to attend to an external target, there is always concern about the extent to which the learner has directed their attention from internal (which is a very powerful attractor for a beginner) to external. Perhaps by simplifying correct execution mechanics through converting it to a metaphor, internal information no longer serves as a powerful attractor to compete with directing attention externally. It appears that in the combined model, learners can more easily direct their attention from movement-related information (internal) to the environment (external) because movement-related information has been reduced due to the use of analogy.

Consistent with this results, Andy & van Ginneken (2017) showed that children’s conscious control propensity moderates the role of attentional focus in motor skill acquisition [67]. Optimization of environmental information in external attentional focus, although limited in the attentional focus literature, does exist. Although research literature has shown the superiority of external over internal attentional focus regardless of age, health conditions, and skill level [68], some studies have sought to answer whether any type of external attentional focus is suitable for performance and learning. For example, Coker (2016) showed that for goal-directed movements requiring maximum effort, personalizing the distance of external attentional focus based on ability enhances its effect [69]. Makaruk et al. (2020) also showed that the combined group (external focus-autonomy support) had fewer unsuccessful shots compared to the external focus or autonomy support groups alone [70]. Their findings indicate that promoting external attentional focus and supporting autonomy can enhance penalty kick performance.

In the present study, there is also the possibility that simultaneous use of external focus and analogical learning models resulted in optimization of the external attentional focus effect. Consistent with the results of this study, one can refer to research that has used combined implicit learning models. Ramezanzade et al. (2022) showed that the errorless-random practice group (both of which are implicit learning models) had better performance compared to other groups in both retention and transfer and automaticity tests [71]. Additionally, in a more related study, Ramezanzade and Doraneh Kord (2018) showed that in the external focus group, there was a significant difference between errorless and errorful pracitce conditions in favor of the errorless group, and the errorless-external focus group performed better than the errorless-internal focus, errorful-external focus, and errorful-internal focus groups in retention and transfer tests [72].

Despite the superior performance of the combined group during acquisition sessions, no significant differences were observed between the combined group and the separate external focus and analogy groups in retention and transfer tests. This pattern—advantage during acquisition but not retention/transfer—can be explained by considering the role **of** instructional scaffolding and working memory demands during self-initiated strategy retrieval. During acquisition, participants in the combined group benefited from ongoing external instructional support, receiving both analogy and external focus cues before each practice block. These externally provided reminders did not require internal generation or maintenance in working memory; participants could immediately apply the cues to their movements. In this scaffolded context, the dual instructions worked synergistically: the analogy simplified movement mechanics into a meaningful metaphor, while the external focus cue optimized attentional allocation away from body movements. Importantly, because these cues were externally provided on each trial, children with limited working memory capacity could benefit from the combined optimization without the burden of retrieving or mentally maintaining complex instructions.

In contrast, retention and transfer tests removed all external instructional cues, requiring participants to self-generate and internally maintain their learned attentional strategies from memory. Here, we propose that the combined instruction created a retrieval burden that was particularly problematic for children with DCD. Research has demonstrated that many developmental disabilities, including developmental coordination disorder, exhibit significant deficits and impairments in visuospatial short-term memory and working memory [73,74]. This suboptimal level of working memory capacity is expected to negatively impact motor learning for children, particularly those with motor difficulties. So, recalling and mentally rehearsing two integrated instructional components (analogy metaphor + external focus target) likely required more working memory resources than recalling a single component. For children with working memory deficits, this retrieval process itself may have consumed cognitive resources that would otherwise be available for movement execution. Single instructions (external focus alone or analogy alone), being simpler to retrieve and maintain, were more robust when external scaffolding was removed. This interpretation is consistent with research demonstrating that children with DCD benefit from external cueing during performance but struggle with self-initiated strategy use when support is withdrawn [74,75]. It also suggests that our relatively brief training period (three acquisition sessions) may have been sufficient for acquisition with external support but insufficient for children with DCD to fully consolidate the complex combined strategy into an easily retrievable, automatic cue. Future research should examine whether extended practice or gradual fading of combined instructions (rather than abrupt removal) allows children with DCD to overcome these retrieval-related working memory demands and demonstrate retention/transfer advantages.

Consistent with these findings, Tse & van Ginneken (2017) demonstrated that the analogy group showed superior performance compared to the explicit instruction group at the beginning of practice, but this difference disappeared during retention [26]. Tse & van Ginneken (2017) trained 5- to 7-year-old children in rope jumping over five lessons during a two-week period. They received either a set of explicit instructions or instructions through analogy. For example, explicit instructions included jumping with both feet at one point. In contrast, children in the analogy learning group were told to “jump like a rabbit.” Both groups showed improvement in successful rope jumping skills, but during early learning (i.e., the first practice session), analogical instructions resulted in more successful jumps and better movement technique than the explicit instruction group. Furthermore, when tested with a concurrent secondary task, children who received analogical instructions demonstrated stronger rope jumping skills. This indicates that performance following learning with analogical instructions is less dependent on cognitive resources than performance following explicit instruction [26].

Based on the results of this research, the performance of implicit groups (analogy, external focus, and combined) in the automaticity test was superior to that of the explicit group (internal focus). The superior dual-task performance observed in the implicit learning groups suggests that these instructions may have resulted in more automatic motor control that is less dependent on cognitive resources. While we interpret this pattern as reflecting reduced working memory demands based on theoretical frameworks [10,33], we acknowledge that working memory capacity was not directly measured in this study, and this interpretation remains speculative. Numerous studies have demonstrated that following practice with implicit methods, performance remains stable under dual-task conditions, whereas performance deteriorates after practice with explicit methods [71,76]. This indicates that performance following implicit methods was indeed more automatic.

Furthermore, the results clearly demonstrated that in groups based on implicit learning, the storage of action-related knowledge in long-term memory (i.e., changes in mental representation structure) was greater compared to the explicit group, indicating improvement in performance as a valid indicator of motor learning [41]. The present findings are consistent with those of Meier et al. (2020) [32]. They examined the effects of analogy learning and explicit instructions on performance and cognitive representations of tennis serve in intermediate-level participants. Results showed that participants in both analogy learning and explicit instruction groups demonstrated greater accuracy over time and more functional mental representation structures. Specifically, the results of the present study showed that the representation structures of participants in implicit groups (analogy, external focus, and combined) were organized in a distinct tree-like hierarchy that was consistent with behavioral results of accuracy in striking in the golf task and had a more organized and structured mental representation structure. The results of this study are consistent with findings from studies examining the effects of various learning variables, such as the effects of observational learning and mental practice on individuals’ mental representation structure [49, 77]. Finally, the results of this study also confirmed the acquisition of implicit knowledge in terms of representation structure, and the results are consistent with the possibility that substantial working memory engagement and extensive conscious processing may not be necessary for acquiring motor patterns during implicit learning, though direct measurement of these cognitive processes would strengthen this conclusion. In this regard, findings from Kim et al.’s (2019) study showed that a triple practice program of action observation (AO) and motor imagery (MI) practice affects learning and mental representations of taekwondo poomsae [60]. These results showed that the simultaneous practice group (AO + MI, AO + MI, …), alternating practice group (AO, MI, AO, MI, …), blocked practice group (AO, AO, …, MI, MI, …), and no-practice group could improve changes in mental representation structure formation and taekwondo poomsae performance. Alternating action observation (AO) and motor imagery (MI) practice combinations may be more effective.

The important point is that although the ARI scores observed in the present study remain modest relative to the expert reference value of 1.0, this gap is consistent with the neurodevelopmental profile of children with Developmental Coordination Disorder. Children with DCD are known to exhibit pervasive deficits in working memory, particularly in the visuospatial domain, which inherently limits their motor performance capacity [75,78]. These cognitive constraints are well-documented and are expected to widen the performance gap between this population and expert benchmarks. Similar ARI ranges have been reported in comparable pediatric DCD populations in previous studies [49,79,80], further supporting the ecological validity of our findings. Therefore, the obtained ARI values should be interpreted within the context of the participants’ developmental limitations rather than as a reflection of methodological inadequacy, and the statistically significant improvement observed nonetheless demonstrates a meaningful treatment effect within this population.

### Limitations and future directions

Several limitations should be acknowledged. First, while working memory is central to our theoretical framework and while our dual-task paradigm provides indirect behavioral evidence consistent with differences in automaticity and cognitive load across instructional conditions, we did not directly measure working memory capacity or working memory demands during motor performance. Therefore, our interpretations regarding reduced working memory load associated with implicit learning methods remain theoretical and should be considered speculative. Future research should incorporate direct measures of working memory (e.g., digit span, spatial span tasks) to examine individual differences in working memory capacity as potential moderators of instructional effectiveness.

Additionally, concurrent measures of cognitive load (e.g., pupillometry, secondary task reaction times with varying difficulty levels, or neurophysiological indices such as EEG) during motor learning would provide more direct evidence for the cognitive mechanisms proposed in this study. Second, our sample consisted of children with DCD aged 7–9 years recruited from specialized motor rehabilitation centers in Tehran, Iran. This limits generalizability in several important ways: (a) findings may not extend to younger or older children with DCD, who may be at different developmental stages and respond differently to instructional manipulations; (b) cultural and educational context may influence how children respond to metaphorical instructions (analogies) and attentional focus cues, limiting generalizability to children in different cultural or linguistic contexts; (c) our findings are specific to children with DCD and cannot be assumed to generalize to typically developing children, who may have different cognitive profiles and learning needs—indeed, as noted in the Discussion, future research should examine whether the combined approach shows similar benefits in typically developing populations; and (d) severity levels within our DCD sample may have varied, and findings may not generalize across the full spectrum of DCD presentations.

Third, the golf putting task, while providing a controlled laboratory measure of aiming and precision skill, represents only one type of motor task and may not generalize to other motor skills with different biomechanical or cognitive demands. More importantly, golf putting is a sport-specific skill rather than a functional activity of daily living. Therefore, caution is warranted in generalizing these findings to the everyday motor challenges that children with DCD face in school, home, and community settings (e.g., handwriting, dressing, ball catching, playground activities). The extent to which improvements in laboratory-based precision tasks translate to meaningful improvements in real-world motor function and participation remains unclear and should be investigated in future ecological validity studies. Future research should address these limitations by: (1) directly assessing working memory capacity and cognitive load during learning, (2) examining the effectiveness of combined implicit instructions in typically developing children to determine whether benefits are specific to populations with working memory deficits, (3) investigating potential mediation or moderation effects of working memory capacity on the relationship between instructional type and motor learning outcomes, and (4) employing neurophysiological measures (e.g., EEG, fMRI) to elucidate the neural mechanisms underlying different instructional approaches in children with DCD.

Our control group received equivalent practice exposure, experimenter contact, and encouragement as experimental groups but no specific attentional or instructional cues. While this design follows established practice in implicit learning research and controls for practice effects and experimenter attention, an alternative design using neutral instructions matched for verbal content and duration (e.g., describing irrelevant aspects of the environment) could provide additional control for potential placebo or expectancy effects. However, such “neutral” instructions risk introducing their own confounds if participants interpret them as meaningful guidance. We believe our approach—providing basic task understanding without strategic guidance—represents an appropriate and conservative control condition, though future research could explore alternative control designs.

## Conclusion

Based on the results of this research and consistent with theoretical frameworks, we tentatively suggest that populations with reduced working memory capacity, such as children with DCD, may benefit more from motor learning patterns that promote implicit learning. However, direct measurement of working memory demands is needed to confirm this mechanism. It appears that in support of analogy learning combined with attentional focus instructions, these two types of practice approaches follow a parallel approach toward strengthening implicit learning, and teaching motor skills through analogy instruction and external attentional focus (simultaneously) can have a multiplicative and additive effect in children with DCD. Based on the results of this research, participants in both analogy learning and explicit instruction groups also demonstrated greater accuracy over time and more functional mental representation structures. These results provide a promising approach to the cognitive mechanism of explicit and implicit practice and can be used for analyzing children’s mental representations during motor skill learning.

## Supporting information

S1 Data The SPSS data file containing the raw dataset used for the statistical analyses reported in this paper. (SAV)
